# Application of Locked Nucleic Acid (LNA) Primer and PCR Clamping by LNA Oligonucleotide to Enhance the Amplification of Internal Transcribed Spacer (ITS) Regions in Investigating the Community Structures of Plant–Associated Fungi

**DOI:** 10.1264/jsme2.ME16085

**Published:** 2016-09-07

**Authors:** Makoto Ikenaga, Masakazu Tabuchi, Tomohiro Kawauchi, Masao Sakai

**Affiliations:** 1 Research Field in Agriculture, Agriculture Fisheries and Veterinary Medicine Area, Kagoshima University1–21–24, Korimoto, Kagoshima, 890–0065Japan; 2 Graduate School of Agriculture, Kagoshima University1–21–24, Korimoto, Kagoshima, 890–0065Japan; 3 The United Graduate School of Agricultural Sciences, Kagoshima University1–21–24, Korimoto, Kagoshima, 890–0065Japan

**Keywords:** plant–associated fungi, community structure, ITS region, LNA primer, PCR clamping by LNA oligonucleotides

## Abstract

The simultaneous extraction of host plant DNA severely limits investigations of the community structures of plant–associated fungi due to the similar homologies of sequences in primer–annealing positions between fungi and host plants. Although fungal-specific primers have been designed, plant DNA continues to be excessively amplified by PCR, resulting in the underestimation of community structures. In order to overcome this limitation, locked nucleic acid (LNA) primers and PCR clamping by LNA oligonucleotides have been applied to enhance the amplification of fungal internal transcribed spacer (ITS) regions. LNA primers were designed by converting DNA into LNA, which is specific to fungi, at the forward primer side. LNA oligonucleotides, the sequences of which are complementary to the host plants, were designed by overlapping a few bases with the annealing position of the reverse primer. Plant-specific DNA was then converted into LNA at the shifted position from the 3′ end of the primer–binding position. PCR using the LNA technique enhanced the amplification of fungal ITS regions, whereas those of the host plants were more likely to be amplified without the LNA technique. A denaturing gradient gel electrophoresis (DGGE) analysis displayed patterns that reached an acceptable level for investigating the community structures of plant–associated fungi using the LNA technique. The sequences of the bands detected using the LNA technique were mostly affiliated with known isolates. However, some sequences showed low similarities, indicating the potential to identify novel fungi. Thus, the application of the LNA technique is considered effective for widening the scope of community analyses of plant–associated fungi.

Plant–associated fungi promote the growth of host plants. Although some fungi have neutral or deleterious effects on plant growth, beneficial fungi protect plants by enhancing resistance to disease and insects (7, 19, 24), solubilizing insoluble soil phosphate (13, 46), supplying available forms of nitrogen (32), and conferring heavy metal and drought tolerance (3, 9). Therefore, investigations on plant–associated fungal community structures provide basic information that facilitates the isolation of beneficial fungi for agricultural applications as microbial materials or bio–fertilizers.

Cultivation methods have been used to characterize fungal communities (11). However, some unisolated and unexploited fungi remain in ecosystems, including those affiliated with plants (12, 14). Hawksworth *et al.* (16) reported that 74K to 120K fungi have been identified to date, whereas 1.5M species were estimated as the total fungal population, indicating that more than 90% of fungal species are still unknown. Fungi that form large colonies on agar plates are preferentially detected in culture–dependent community analyses, resulting in the underestimation of other fungal community components (12). However, a culture–independent molecular technique is more advantageous for studying fungal communities because it provides information on differences between community structures irrespective of culturable, unisolated, and unexploited fungi. This approach has accelerated community analyses of plant–associated fungi.

In community analyses using culture–independent molecular techniques, small subunit (SSU) and large subunit (LSU) rRNA genes and the internal transcribed spacer (ITS) region are frequently used as barcoding sequences (17, 30, 42, 43, 49, 59). The ITS region has been identified as a suitable barcoding region for a fungal community analysis because its sequences vary more than those of SSU and LSU rRNA genes (45, 47). However, previous community analyses of plant–associated fungi have frequently been limited due to contamination by host plant DNA (15, 18). This has been attributed to the regions of the SSU and LSU rRNA genes used in the design of primer sets to amplify the fungal ITS region, a sequence that is almost homologous to those found in host plant DNA (51), and results in an abundance of host plant amplicons in PCR products. This unspecific amplification has delayed understanding of the roles of fungi in plant growth. In response to this major limitation, fungal-specific primers were designed to amplify the ITS regions (15, 35). However, these primers had low coverage for fungi, resulting in a PCR amplification bias (6, 51).

A locked nucleic acid (LNA) is an artificial nucleotide analog that contains a methylene bridge connecting the 2′–oxygen of ribose with the 4′–carbon (26, 39). This bridge results in a locked 3′–*endo* conformation with reduced conformational flexibility (29, 33). The LNA base may be incorporated into oligonucleotide sequences similar to a DNA base (8, 25). The incorporated LNA oligonucleotide (LNA oligonucleotide) shows extraordinary mismatch sensitivity to complementary nucleic acids in LNA/DNA hybrids (26, 29), and higher thermal stability than DNA oligonucleotides (48, 55).

We have developed an LNA oligonucleotide–PCR clamping technique in order to investigate the community structures of plant–associated bacteria (20, 21, 22). This technique has enabled the PCR amplification of bacterial SSU rRNA genes, while inhibiting the amplification of host plant organelle (mitochondria and plastid) genes. Although this innovative technique is considered to be applicable to the selective amplification of fungal ITS regions, it is still limited because the ITS regions of other DNA derived from microeukaryotes, such as protozoa, as well as those of the host plant are also amplified due to mismatches in fungal-specific primers during PCR (15, 35). The PCR clamping technique may be employed when DNA sequences expected to be inhibited are already known; however, this technique is insufficient when DNA sequences are unspecified, such as in eukaryotic communities. In this case, the application of a fungal-specific primer incorporating LNA bases (an LNA primer) is suitable for avoiding mismatches in primer annealing for the selective amplification of fungal ITS regions on the forward and reverse sides. Alternatively, the combination of LNA primers and the LNA oligonucleotide–PCR clamping technique, in which LNA oligonucleotides are specific for host plant DNA predominantly contaminating extracted DNA, appears to be effective.

In order to develop this new method for a fungal community analysis, we selected wheat, soybean, and potato as representative plants, and targeted *Ascomycota* and *Basidiomycota*, which account for approximately 80% of all fungus species currently identified (6), together with the known arbuscular mycorrhizas, *Glomeromycota*. We then tested the effectiveness of the LNA technique in a community analysis of plant–associated fungi by comparing the DGGE patterns generated with and without the LNA technique. We also examined possible interference by LNA oligonucleotides during the PCR amplification of fungal ITS regions using DNA extracted from agricultural field soil.

## Materials and Methods

### Preparation of plant samples

Wheat (*Triticum aestivum* ‘Chikugoizumi’), soybean (*Glycine max* ‘Fukuyutaka’), and potato (*Solanum tuberosum* ‘Nishiyutaka’) were cultivated under upland conditions. In brief, brown lowland soil was collected from the plow layer of an agricultural field at Kagoshima University (latitude 31°34′ N, longitude 130°32′ E), and placed into 1/5000a pots. After germination, wheat and soybean were cultivated in the potted soils for several days. Twenty wheat and soybean seedlings were harvested, and the roots were collected separately. Potato seed tubers were directly transplanted into the pots and cultivated for one month. The leaves, stems, roots, and epidermises from secondary young tubers were separately collected from five individuals. The epidermises of mother tubers were also collected, and all samples were ground for DNA extraction and adjusted to 0.5 g fresh weight mL^−1^ with sterilized water.

In order to prepare control DNAs, which only produce amplicons of host plant ITS regions after PCR, the aseptic roots of wheat and soybean were prepared according to the protocol specified by Sakai and Ikenaga (44) using 1/10 tryptic soy agar (34). A small section of the inner tuber of the potato was aseptically collected. These samples were ground and adjusted following the same procedure, and regarded as aseptic samples.

### DNA extraction from plant samples and soils

Approximately 0.4 g (ground and adjusted weight) of roots from wheat and soybean, and the leaves, stems, roots, and epidermises of young and mother tubers from potato were used for DNA extraction in duplicate by ISOPLANT II (Nippon Gene, Toyama, Japan) with the bead–beating method. The same amounts of aseptic samples prepared from wheat, soybean, and potato were used in the extraction. In brief, samples were physically shaken by bead–beating at 4,800 rpm for 60 s using MS–100 TOMY (TOMY, Tokyo, Japan), then chemically treated with benzyl chloride. Subsequent procedures followed the manufacturer’s instructions, with minor modifications.

Approximately 0.5 g fresh soil collected from the agricultural field in Kagoshima University was used for DNA extraction. Extraction was performed using the FastDNA SPIN Kit for Soil (MP Biomedicals, Solon OH, USA) according to the manufacturer’s instructions. Following the extraction of plant and soil samples, crude DNA was purified using the Power Clean DNA Clean UP Kit (MO BIO, Carlsbad, CA, USA).

### Designing LNA primers and LNA oligonucleotides

The fungal and plant (wheat, soybean and potato) sequences of SSU rRNA and LSU rRNA genes, located upstream and downstream of the ITS region, respectively, were obtained from the nucleotide database of the National Center for Biotechnology Information (NCBI; http://www.ncbi.nlm.nih.gov). Fungal sequences were collected from the three major phyla *Ascomycota*, *Basidiomycota*, and *Glomeromycota*, which contain the sequences of plant–associated fungi.

Sequences were aligned with the primers used to amplify fungal ITS regions by CLUSTAL W version 1.7 (50), and the relevant sequences were organized by removing gaps using BioEdit (http://www.mbio.ncsu.edu/bioedit/bioedit.html). The possible sequences to design LNA primers specific to fungi or LNA oligonucleotides specific to plants for application to the clamping technique were examined in consideration of primer coverage.

Based on the aligned sequences, LNA primers were designed by converting the fungal-specific DNA base into an LNA base in order to enhance its specificity to fungi. LNA oligonucleotides were designed by overlapping a few DNA bases with the annealing position of the fungal primer at the extension side, and converting DNA bases, which were only specific to plants, into LNA bases at the shifted position. The 3′ ends of LNA oligonucleotides were phosphorylated to avoid extension during PCR. The sequences of LNA oligonucleotides were verified using the BLAST search program (http://blast.ncbi.nlm.nih.gov/Blast.cgi) (1) to confirm if identical sequences existed in fungi.

### Estimation of the *Tm* values of LNA oligonucleotides

In order to estimate the *Tm* values of LNA oligonucleotides, PCR amplification was performed at different annealing temperatures using a PCR thermal cycler (PC350, ASTEC, Fukuoka, Japan). DNA extracted from wheat and soybean roots was used as representative samples. The PCR mixture contained *Premix* Hot Start Version (Takara, Otsu, Japan), the fungal primer set (0.8 μM each), and the DNA template. PCR conditions were as follows: 94°C for 3 min (initial denaturation), followed by 40 cycles at 94°C for 1 min, annealing from 60°C to 74°C at 2°C intervals for 1 min, and 72°C for 2 min, with a final extension step at 72°C for 10 min. Aliquots of the PCR products were electrophoresed after amplification. The *Tm* values of the LNA oligonucleotides were estimated from amplicon intensities.

### Estimation of effective concentrations of LNA oligonucleotides

The extracted DNA of the respective samples was used in the amplification to estimate the effective concentrations of LNA oligonucleotides. The PCR mixtures contained serial concentrations of LNA oligonucleotides, and comprised 0 μM, 0.5 μM, 1.0 μM, 2.0 μM, 3.0 μM, and 4.0 μM. Mixtures of 6.0 μM and 8.0 μM were also prepared for potato leaf and stem samples. PCR tubes contained extracted DNA, forward and reverse primers (0.8 μM each as the final concentration), *premix Ex Taq* Hot Start Version (Takara), an LNA oligonucleotide (0 μM to 8.0 μM as the final concentration), and sterilized ultra-pure water. An LNA primer or DNA primer was used as a forward primer. PCR tubes, containing extracted DNA from the respective samples and aseptic samples, were also prepared without using the LNA technique.

In order to apply PCR clamping by LNA oligonucleotides, the annealing step for the LNA oligonucleotides was added between the denaturation step and annealing step of the fungal primers during the amplification process. A 1-min incubation period was added between the steps. The program implemented the following steps: 94°C for 3 min (initial denaturation), followed by 40 cycles at 94°C for 1 min, annealing of the LNA oligonucleotide for 1 min, 54°C for 1 min (annealing step of fungal primers) and 72°C for 2 min, with a final extension step at 72°C for 10 min. Aliquots of the PCR products were electrophoresed, and the effective concentrations of the LNA oligonucleotides were estimated by confirming the product trend, which exhibited the same mobility as the amplicons of aseptic samples.

### Nested PCR amplification and DGGE

The PCR products obtained under optimized conditions were again amplified using the fungal primer set for denaturant gradient gel electrophoresis (DGGE). Prior to nested PCR, the products were purified and serially diluted 10^3^- to 10^4^-fold. The products amplified without the LNA technique were also prepared to compare DGGE patterns. In addition, extracted DNA from aseptic roots was directly amplified with the primer set used for DGGE in order to confirm the DGGE banding positions of host plants.

DGGE was performed in a gel containing a linear chemical gradient ranging from 20% to 60% of the denaturant (37). Approximately 600 ng of amplicons were loaded and electrophoresed at 60°C and 100 V for 14 h using a DCode universal mutation detection system (BioRad Laboratories, Hercules CA, USA). After electrophoresis, the gel was stained with SYBR gold (Life Technologies Japan, Tokyo, Japan) and photographed under UV illumination.

### Sequencing of DGGE bands

The sequences of some DGGE bands, which were newly detected using the LNA technique, were obtained by direct sequencing or TA cloning–assisted sequencing.

In direct sequencing, the bands were excised from the gel and directly amplified as a DNA template. A mobility check of the amplified band was performed to confirm whether the position of the band was the same as that of the original by replicating the DGGE analysis under identical conditions. A forward primer without the GC clamp and a reverse primer for DGGE were used for cycle sequencing. DNA sequencing was performed using the ABI 3500*xL* Genetic Analyzer (Life Technologies Japan).

In TA cloning–assisted sequencing, the amplicons used for DGGE were purified and ligated to the pT7 Blue T–vector (Novagen, Madison, WI, USA) with TA–Blunt Ligation Kit (Nippon Gene). The ligated products (plasmids) were transformed in competent cells of *ECOS* Competent *E. coli* XL1–Blue (Nippon Gene). After blue/white selection, the DGGE bands incorporated as inserts in the plasmid were amplified with the T7 primer (5′–TAATACGACTCA CTATAGG–3′) and U–19 primer (5′–GTTTTCCCAGTCACGAC GT–3′), and nested PCR was performed for the purified products with the primer set used for DGGE. A mobility check of the products was performed by DGGE in order to confirm the positions of the bands. The T7 and U–19 primers used for cycle sequencing corresponded to the purified T7 and U–19 products

### Examination of possible interference by LNA oligonucleotides during PCR for the amplification of fungal DNA

The extracted DNA from agricultural field soil was amplified to examine the possible interference of LNA oligonucleotides in the amplification of fungal ITS regions during PCR. A reaction without LNA oligonucleotides was also conducted as a control. DGGE was performed in an acrylamide gel containing a linear chemical gradient ranging between 20% and 60%. The patterns generated with and without LNA oligonucleotides were compared.

### Nucleotide sequence accession number

The sequences obtained in this study are available on DNA databases under the accession numbers LC0262298–LC026315.

## Results and Discussion

### Investigation of designable regions for LNA primers and LNA oligonucleotides

The forward and reverse primers used in the fungal community analysis were collected in order to identify designable regions for LNA primers and LNA oligonucleotides. The forward primers were ITS9mun (10), NSI1 (36), ITS1F (15), ITS1F_KYO1 and ITS1F_KYO2 (51), ITS5 (57), and ITS1 (57). The reverse primers were ITS4 (57), ITS4_KYO1, ITS4_KYO2, and ITS4_KYO3 (51), ITS8mun (10), NLB4 (36), and NLC3 (36). The forward and reverse primers were designed to hybridize downstream of 18S rRNA genes and upstream of 26S rRNA genes, respectively, in order to amplify fungal ITS regions for the community analysis.

Alignment was performed using the sequences of fungi derived from *Ascomycota*, *Basidiomycota*, and *Glomeromycota*, and of agricultural plants including wheat, soybean, and potato, together with primer sequences. After alignment on the forward side, LNA primers were only designed for the annealing positions of the NSI1 and ITS1F primers, while the positions to design LNA oligonucleotides for PCR clamping were not observed on the forward side (data not shown). When comparing fungal sequence coverage, the NSI1 primer had 77.9% coverage in the phyla *Ascomycota*, *Basidiomycota*, and *Glomeromycota*, while higher coverage (89.8%) was achieved by the ITS1F primer (51). In addition, the ITS1F primer was designed to contain specific bases to enhance the selective amplification of fungal DNA (15). These results suggest that the ITS1F primer is optimal for designing LNA primers by replacing specific bases with LNA bases.

On the other hand, the positions to design LNA primers were not observed on the reverse side (data not shown). However, a modifiable position for LNA oligonucleotides was found to be in competition with the ITS4 primer. Although the ITS4 primer has been designed to cover whole eukaryotes (57), specific bases for plants were found in a position that was shifted toward the extension side from the annealing position. Host plant DNA was generally abundant in extracts from plant samples. It is reasonable to inhibit its amplification by applying the LNA oligonucleotide–PCR clamping technique. As a result, the combination of an LNA primer on the forward side and the clamping technique on the reverse side was considered to be effective for enhancing the PCR amplification of the fungal ITS region. The primer set ITS1F and ITS4 has also frequently been used to amplify the fungal ITS region in environmental samples (2, 5, 27, 41, 56).

### Designing LNA primers

**Fig. 1:**
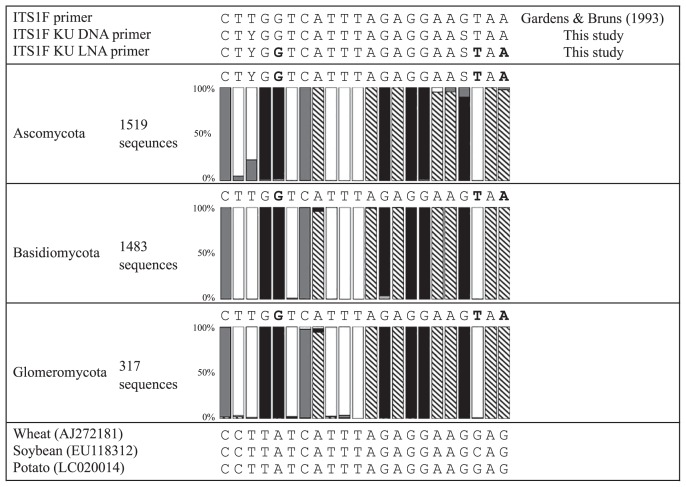
Alignment data of fungal and plant sequences to design ITS1F KU DNA and ITS1F KU LNA primers specific for fungi. The symbols 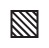, □, ■, and 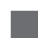 in the columns indicate the DNA bases of A, T, G, and C, respectively. The bases indicated with bold letter bases are specific bases for fungi. These DNA bases were replaced with LNA bases.

Fig. 1 shows the alignment sequences of fungi and representative agricultural plants, including wheat, soybean and potato, on the forward side together with the ITS1F primer. The numbers of fungal sequences used in the alignment were 1,519, 1,483, and 317 as derived from *Ascomycota*, *Basidiomycota*, and *Glomeromycota*, respectively. These were not the numbers of species, but those of fungal sequences that were widely collected in consideration of phylogenetic diversities.

The 3rd and 19th bases from the 5′ end were mostly T and G for the three fungal phyla. However, C was found at a certain percentage in both bases in *Ascomycota*. In order to increase the coverage of the ITS1F primer, these bases were degenerated as Y (T or C) and S (C or G) in the 3rd and 19th bases, respectively. The modified primer was named the ITS1F KU DNA primer, and its sequence was 5′–CTYGGTCATTTAGAGGAASTAA–3′.

In addition, the 5th, 20th, and 22nd bases from the 5′ end were G, T, and A in fungal sequences, while the corresponding bases were A, C, and G in plant sequences, indicating the availability of DNA bases to be converted to LNA bases in a fungal–specific LNA primer design. The LNA primer designed was named the ITS1F KU LNA primer, and its sequence was 5′–CTYGGTCATTTAGAGGAASTAA–3′ (underlined bases indicated the LNA bases).

### Estimation of *Tm* values for LNA oligonucleotides

In order to apply the PCR clamping technique, the annealing step of LNA oligonucleotides needs to be added between the denaturation step and annealing step of fungal primers, which inhibits the amplification of host plant DNA by LNA oligonucleotides. In this step, the annealing temperature for LNA oligonucleotides needs to be higher than that for the fungal primer, at which fungal primers are non–functional, thereby avoiding hybridization with host plant DNA. If fungal primers hybridize at the higher temperature, host plant DNA is easily amplified during PCR for excessive inclusion in DNA extracts. In addition, the annealing temperature for LNA oligonucleotides needs to be as low as possible. If the temperature is too high, the efficiency of hybridization for LNA oligonucleotides is decreased, resulting in the amplification of host plant DNA in the subsequent step. In order to avoid the hybridization of the ITS4 primer at the corresponding position of host plant DNA during the annealing step of LNA oligonucleotides, the *Tm* values of LNA oligonucleotides were estimated by PCR using the ITS1F KU LNA and ITS4 primers.

**Fig. 2:**
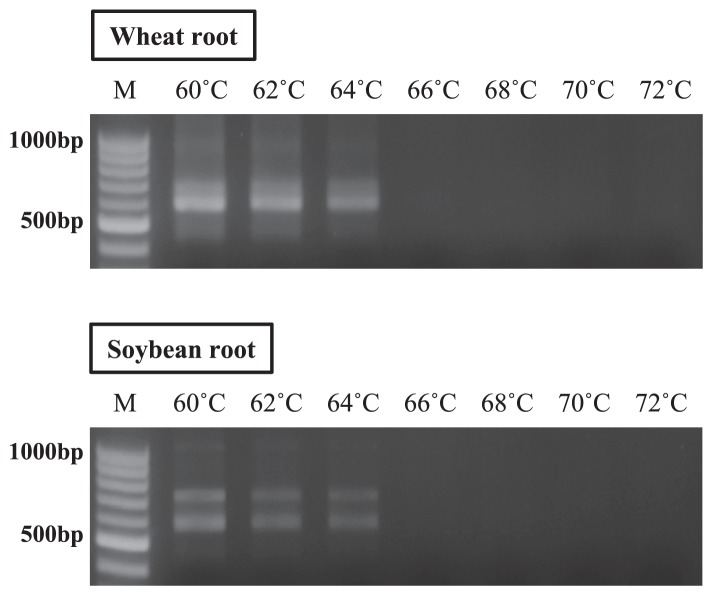
Estimation of *Tm* values for LNA oligonucleotides. The annealing temperatures at which fungal primers were non–functional were used for the *Tm* values of LNA oligonucleotides. Annealing temperatures ranged between 60°C and 72°C, with increments of 2°C. “M” is the marker for the 100-bp ladders.

As shown in Fig. 2, the amplification products of DNA extracted from wheat and soybean roots were detected at high intensities at 60°C. However, their intensities decreased until 64°C with an increase in the annealing temperature, and no products were detected at temperatures higher than 66°C. This result indicated that the primer set was functional up to 64°C; however, this temperature appeared to be high because the *Tm* values of the ITS1F KU LNA and ITS4 primers were 59–62°C and 60°C, respectively. Therefore, the high temperature resulted in low product intensities after PCR amplification. Based on the results of electrophoresis, the *Tm* values of LNA oligonucleotides were estimated to be approximately 70°C in order to avoid the annealing of fungal primers.

### Designing ITS4 LNA oligonucleotides

**Fig. 3:**
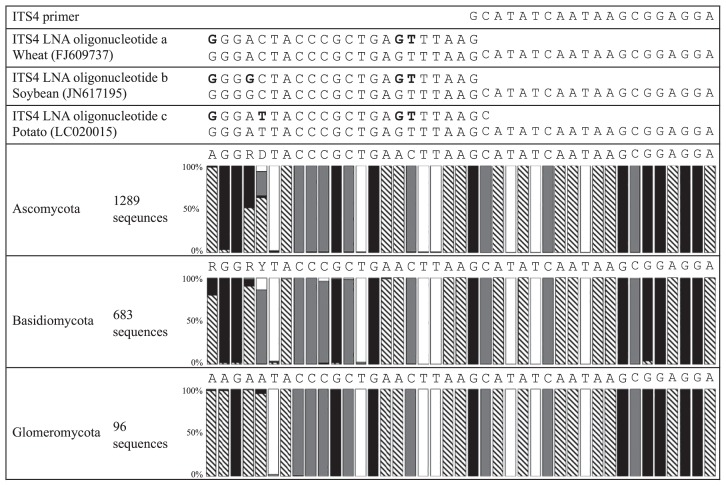
Alignment data of plant and fungal sequences to design ITS4 LNA oligonucleotides specific for plants. Three types of ITS4 LNA oligonucleotides were designed based on host plant sequences. They were named ITS4 LNA oligonucleotides a, b and c, respectively. The symbols 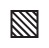, □, ■, and 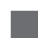 in the columns indicate the DNA bases of A, T, G, and C, respectively. The bases indicated with bold letter bases are specific bases for the host plants. These DNA bases were replaced with LNA bases.

Fig. 3 shows the alignment sequences of representative agricultural plants, including wheat, soybean, and potato, and fungi from three phyla on the reverse side to design LNA oligonucleotides that compete with the ITS4 primer at the annealing position. In this design, the numbers of fungal sequences used in the alignment were 1,289, 683, and 96, derived from *Ascomycota*, *Basidiomycota*, and *Glomeromycota*, respectively. These were also the numbers of fungal sequences widely collected in consideration of diversities.

After alignment, wheat, soybean, and potato each had individual sequences in the LNA oligonucleotide position. The other representative plants were grouped into one of the three types: a) rice, maize, melon, and carrot, b) spinach, peanut, and banana and c) thale cress, tomato, sweet potato, coffee, orange, cotton, and jute. These groups showed the same sequences as wheat, soybean, and potato, respectively. The sequence types of other agricultural plants are listed in Supplementary Table S1. Based on the alignment in Fig. 3, three LNA oligonucleotides specific for wheat (type a), soybean (type b), and potato (type c) were designed by overlapping one or two bases with the 3′ end of the ITS4 primer, and by replacing specific bases for plants with LNA bases in order to equally distribute LNA bases throughout the sequences, with the exception of the overlapped base with the ITS4 primer. Simultaneously, *Tm* values were regulated at approximately 70°C using the automatic calculator on the EXIQON website (https://www.exiqon.com/ls). The LNA oligonucleotides designed were named ITS4 LNA oligonucleotides a, b, and c, respectively. The *Tm* values of ITS4 LNA oligonucleotides a, b, and c were 68°C, 72°C, and 69°C, and their sequences were 5′–**C**TTAAACTCAGCGGGTAGTCCCp–3′, 5′–**C**TTAAACTCAGCGGGTAGCCCCp–3′, and 5′–**GC**TT AAACTCAGCGGGTAATCCCp–3′, respectively. The bold bases indicate the bases overlapping with the ITS4 primer, and the LNA bases are underlined. The 3′ end was phosphorylated (indicated as p) to avoid extension from the oligonucleotides during PCR.

The BLAST search program was used to investigate whether the three oligonucleotides were identical to fungal sequences. LNA oligonucleotides a, b, and c completely matched 7, 1, and 6 fungal sequences, respectively. They were mostly affiliated with uncultured fungal clones. Owing to this negligible amount, the LNA oligonucleotides designed were considered to be available for PCR clamping in order to inhibit the amplification of host plant DNA.

### Estimation of effective concentrations of LNA oligonucleotides

**Fig. 4:**
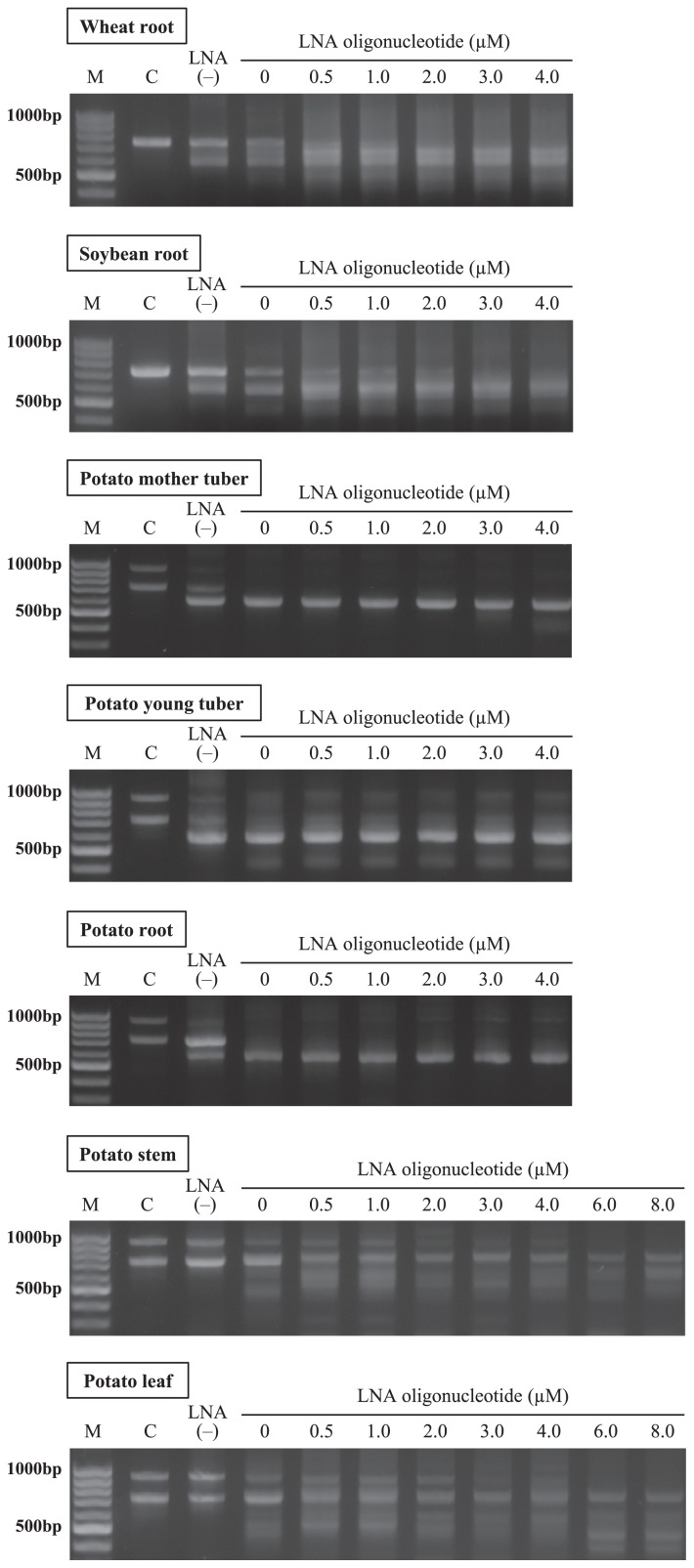
Estimation of effective concentrations for LNA oligonucleotides. LNA oligonucleotides were used in the ranges of 0 μM, 0.5 μM, 1.0 μM, 2.0 μM, 3.0 μM, and 4.0 μM. Higher concentrations of 6.0 μM and 8.0 μM were also examined for potato leaf and stem samples. “M” is the marker for 100-bp ladders, and “C” is aseptic amplicons to provide the position of host plant DNA in the agarose gel. “LNA(−)” represents amplicons prepared with the ITS1F DNA primer and ITS4 primer.

Effective concentrations of the LNA oligonucleotides were examined using 0 μM, 0.5 μM, 1.0 μM, 2.0 μM, 3.0 μM, and 4.0 μM to extracted DNA from the respective plant samples (Fig. 4). Higher concentrations of oligonucleotides, namely, 6.0 μM and 8.0 μM, were also examined with those of potato leaf and stem samples. ITS1F KU LNA and ITS4 primers were used as forward and reverse primers, respectively. In addition, two different amplicons were prepared; one was the product amplified using ITS1F KU DNA and ITS4 primers using DNA extracted from aseptic samples (Lane C). One was used to confirm the mobility locations of host plant products in agarose and DGGE gels, while the other, which was shown as LNA(−), was the product amplified with the ITS1F KU DNA and ITS4 primers using DNA extracted from the respective samples to produce amplicons prepared without LNA primers or PCR clamping by LNA oligonucleotides.

As shown in Fig. 4, the products detected in aseptic samples (Lane C) were also observed in Lane LNA(−) in all samples examined. Bands with the same mobility exhibited high intensity for wheat and soybean roots and the potato leaf and stem. This was presumably caused by the low fungal DNA/plant DNA ratio in the DNA extracted. Consequently, host plant DNAs were excessively amplified by the mismatch of the primer, even the ITS1F KU DNA primer, which had been designed specifically for fungi. However, when the ITS1F KU LNA primer was applied to these samples, the intensities of the host plant products decreased and the other bands with different sizes were additionally detected and/or their intensities increased (Lane 0 μM).

In contrast, the root and epidermises of young and mother potato tubers showed no bands of the same mobility as the aseptic samples when the ITS1F KU LNA primer was used in amplification, while other bands with different sizes were predominantly detected. This result suggests that fungal ITS regions are selectively amplified using the LNA primer only in the samples, which was considered to have a high fungal DNA/plant DNA ratio. Although faint bands giving similar locations to those of aseptic potato products were still detected in the epidermis of the young tuber (Lane 0 μM), they were imprecise. Fungal ITS regions are known to show different lengths, from approximately 400 bp to 900 bp (51); therefore, these bands were assumed to be fungal ITS products.

Thus, the application of an LNA primer enhanced the amplification of the ITS regions of plant–associated fungi more than a DNA primer. However, amplicons of host plant DNA were still detected at small intensities in samples that were considered to have a low fungal DNA/plant DNA ratio. In order to further inhibit the amplification of host plant DNA, the LNA oligonucleotide–PCR clamping technique was applied to compete with the ITS4 primer. LNA oligonucleotides a, b and c were used for wheat, soybean, and potato samples, respectively. As a result, the band intensities derived from host plants decreased with increases in the concentration of LNA oligonucleotides. No bands were observed at more than 2.0 μM, 3.0 μM, 6.0 μM, and 6.0 μM for the wheat root, soybean root, potato leaf, and potato stem, respectively, indicating that the combination of an LNA primer and the LNA oligonucleotide–PCR clamping technique effectively enhanced the amplification of fungal DNA, while inhibiting the amplification of host plant DNA.

### Effects of the LNA technique in investigations of community structures of plant–associated fungi

The fungal PCR products that were selectively amplified using either an LNA primer or LNA primer and PCR clamping by LNA oligonucleotides were used for a fungal community analysis via DGGE in order to compare the patterns amplified by the conventional approach using ITS1F KU DNA and ITS4 primers. Nested PCR products were prepared from these products with the ITS1F KU DNA primer with the GC clamp (5′–CGCCCGCCGCGCGCGGCGGGCGGGGCGG GGGCACGGGGGGCTYGGTCATTTAGAGGAASTA A–3′, the underlined sequence indicates the GC clamp) and ITS2 primer (5′–GCTGCGTTCTTCATCGATGC–3′). The nested products were then used in the DGGE analysis to examine the effects of the LNA technique by comparing these patterns. In addition, DNA extracted from aseptic samples was directly amplified with the ITS1F KU DNA primer with the GC clamp and ITS2 primer in order to confirm the position of host plant amplicons in the DGGE gel. The ITS1F primer set with the GC clamp and ITS2 has frequently used been in community analyses of fungi in various environments (2, 5), while the ITS1F sequence was modified to increase coverage in this study.

**Fig. 5:**
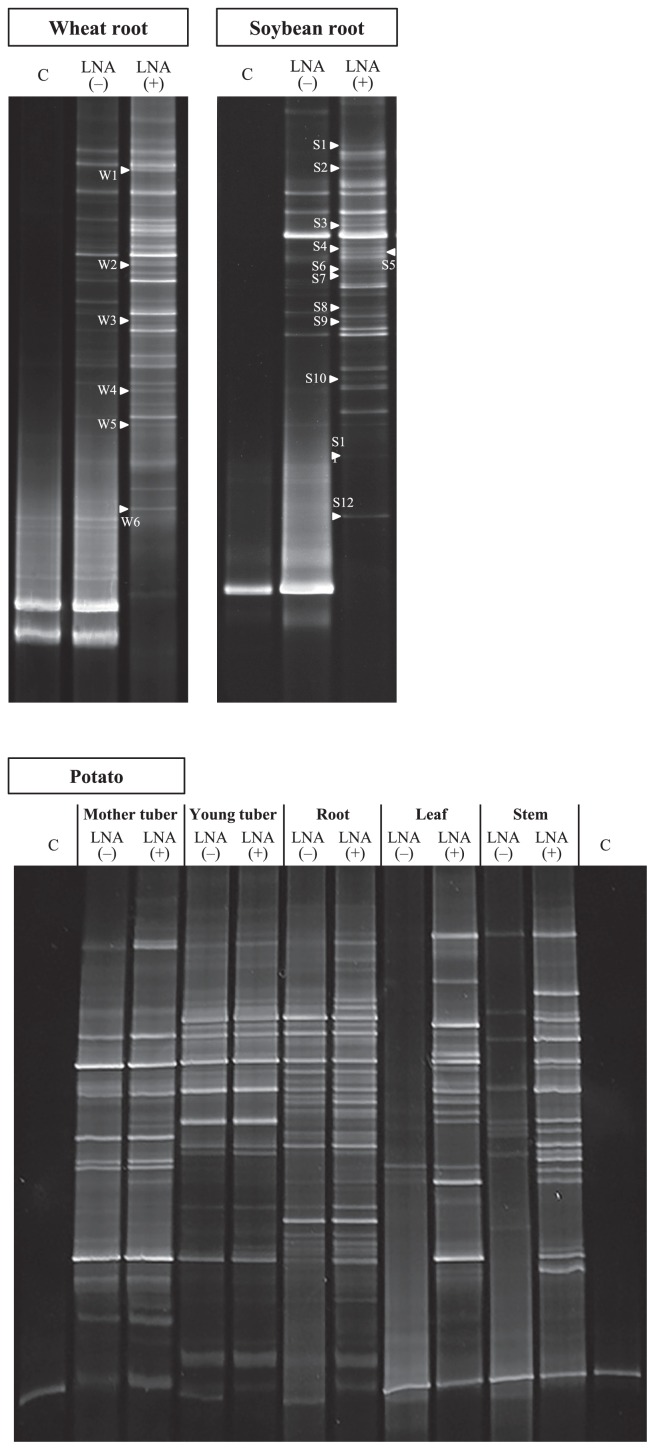
DGGE patterns of nested PCR products derived from respective parts of wheat, soybean, and potato samples. The products were prepared with the ITS1F KU DNA primer with the GC clamp and ITS2 primer. The symbols “−” and “+” indicate the lanes prepared without and with the LNA technique. The products that sufficiently amplified the fungal ITS regions are used in lane “+”. “C” represents aseptic amplicons to provide the position of the host plant band in the DGGE gel. The sequences of DGGE bands indicated with arrows were obtained in order to identify the closest relatives using the DNA database.

As shown in Fig. 5, predominant DGGE bands giving the same mobility as aseptic samples were detected in wheat and soybean roots and in the potato leaf and stem amplicons prepared by the conventional approach without the LNA technique; however, several other bands derived from fungi were also observed in these patterns. This result indicated that the conventional approach underestimated the community structures of plant–associated fungi, particularly in samples with a low fungal DNA/plant DNA ratio.

In contrast, when the LNA technique was applied, DGGE bands showing the same mobility as the host plants were not detected in the patterns of wheat and soybean roots, or the band intensities of host plants were significantly decreased in those of the potato leaf and stem. Furthermore, fungal DGGE bands were additionally detected at an acceptable level to investigate community structures. This was most likely due to fungal bands, which were hidden behind more strongly amplified host plant DNA, becoming visible on DGGE images.

In contrast to that described above, DGGE patterns generated from the roots and epidermises of young and mother potato tubers showed almost identical patterns between LNA(−) and LNA(+). These patterns only differed due to several bands showing greater intensities in the LNA(+) patterns than those in the LNA(−) patterns, and the band derived from host plant DNA was detected in the LNA(−) patterns. This result suggests the potential to investigate the community structures of plant–associated fungi without applying LNA primers when the host plant DNA band is faint. However, it is preferable to completely inhibit the amplification of host plant DNA by applying the LNA technique in order to obtain more accurate information.

### Closest relatives of DGGE bands detected by applying the LNA technique

Some DGGE bands, newly detected using the LNA primer and PCR clamping by LNA oligonucleotides, were sequenced for wheat and soybean roots. As shown in Table 1, all of the closest relatives belonged to fungi, and 15, 2, and 1 sequences were affiliated with *Ascomycota*, *Basidiomycota*, and unidentified fungi, respectively.

Of these, the closest relatives of bands W1, W2, W3, W4, S2, S4, S7, S8, S9, and S10 and W6, S1, and S12 were isolated from plants and agricultural soils, respectively. The closest relatives of bands W1, W3, W4, S8, and S11 are known to be fungi associated with wheat and soybean (4, 23, 28, 40, 52, 54). *Phoma* sp. (closest relatives of bands W3 and S8) and *Acremonium* sp. (closest relatives of band S11) were reported to be beneficial for host plants through their roles in growth promotion and enhancements in resistance to insects and antimicrobial activity, respectively (7, 38, 58). The closest relatives of W2, W4, W6, S9, and S12 have not yet been isolated from wheat and soybean; however, *Acidomelania panicicola* (closest relative of band W2), *Podospora glutinans* (closest relative of band W4), and *Scolecobasidium terreum* (closest relatives of bands W6 and S12) were in the group of dark septate endophytic fungi (32, 53, 60) that contribute to plant growth in a number of ways (31).

Thus, the LNA technique–assisted community analysis is an effective method to obtain genetic information on plant– associated fungi, for which DNA had been screened by the conventional amplification of host plants DNA. This provides fundamental information for investigating the roles of fungi in plant growth, and subsequently to isolate fungi for further investigations, including the evaluation of microbial materials for agricultural applications.

### Examination of possible interference by LNA oligonucleotides during PCR for the amplification of fungal DNA

The LNA oligonucleotides designed effectively amplify fungal DNA while suppressing the amplification of host plant DNA during PCR. However, LNA oligonucleotides may hybridize fungal DNA, the sequences of which are almost identical to host plant DNA. Consequently, the amplification of fungal DNA is inhibited, and the resultant DGGE patterns of plant–associated fungi may be affected. In order to investigate possible interference by LNA oligonucleotides in the amplification of fungal ITS regions, agricultural field soil, which contained an unspecified large variety of fungal DNA, was regarded as a sample, and DGGE patterns were compared between the amplicons prepared with and without LNA oligonucleotides (a, b, and c; added at 4.0 μM each). In the present study, PCR amplification was performed using ITS1F KU with the GC clamp, and ITS 2 after amplifying the whole ITS region with the ITS1F KU LNA primer, and ITS4 for extracted DNA.

**Fig. 6:**
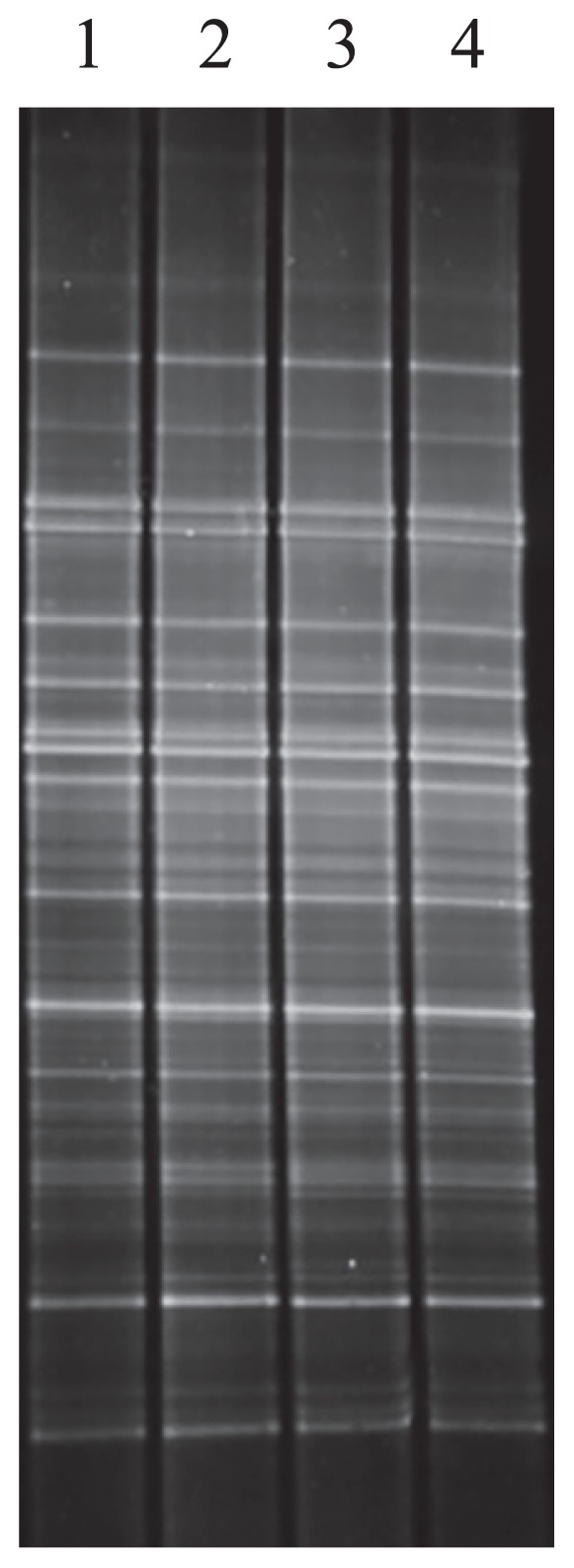
DGGE patterns of nested PCR products derived from agricultural soil. Products were prepared with the ITS1F KU DNA primer with the GC clamp and ITS2 primer after having amplified the whole ITS region with the ITS1F KU LNA primer and ITS4 primer for extracted DNA. LNA oligonucleotides a, b, and c were added to amplify the whole ITS region in order to examine possible interference in the amplification of fungal ITS regions during PCR. Lane 1 indicates the pattern prepared without LNA oligonucleotides, while lanes 2, 3, and 4 indicate those prepared with LNA oligonucleotides a, b, and c, respectively.

As shown in Fig. 6, the soil fungal DGGE patterns among lanes 1 to 4 were identical irrespective of the addition and types of LNA oligonucleotides. This result indicates that LNA oligonucleotides did not affect the amplification of fungal ITS regions, or, if they did, it was negligible for an investigation of fungal communities using a DGGE analysis.

## Conclusion

LNA primers and PCR clamping by LNA oligonucleotides were applied to enhance the amplification of fungal ITS regions for investigations of plant–associated community structures using DNA extracted from plant samples containing eukaryotic DNA, particularly host plant DNA. Overall, the application of LNA primers enhanced the amplification of fungal DNA. LNA primers sufficiently amplified the fungal ITS region in samples, presumably with a high fungal DNA/plant DNA ratio. On the other hand, the combination of LNA primers and PCR clamping by LNA oligonucleotides was necessary for samples with a low fungal DNA/plant DNA ratio. A DGGE analysis showed that fungal banding patterns reached an acceptable level for investigating the community structures of plant–associated fungi. The closest relatives of the DGGE bands all belonged to fungi and some were assumed to contribute to plant growth in a number of ways. Thus, the LNA technique assisted in a fungal community analysis of plant–associated fungi. This amplification technique will benefit further investigations.

## Supplementary Information



## Figures and Tables

**Table 1:** Closest relatives of DGGE bands that were detected using LNA technique

DGGE band	Closest relatives	Phylum	Accession number	Similarity	Seq (bp)	Alignment
W1	*Fusarium oxysporum* GSJY8	Ascomycota	KM457088	99%	192	191/192
W2	*Acidomelania panicicola* 61R41	Ascomycota	KF874617	99%	193	167/169
W3	*Phoma* sp. 659 AI-2013	Ascomycota	KC662225	99%	179	179/181
W4	*Podospora glutinans* F1	Ascomycota	HG971763	94%	219	204/218
W5	*Acanthostigma perpusillum* UAMH 7237	Ascomycota	AY916492	86%	217	190/220
W6	*Scolecobasidium terreum* KNU9	Ascomycota	KJ921609	99%	303	294/296

S1	Uncultured fungus clone 107A62543	Unidentified	JX328731	98%	273	268/273
S2	*Paraphoma* sp. L13	Ascomycota	FJ903342	96%	246	236/246
S3	*Pseudogymnoascus* sp. G AM-2013	Ascomycota	KF039897	100%	248	248/248
S4	*Microporus flabelliformis* Dai11574	Basidiomycota	JX569740	99%	276	222/225
S5	*Aphanoascus fulvescens* NBRC 30411	Ascomycota	JN943432	100%	282	268/268
S6	*Lecythophora* sp. NG_p46	Ascomycota	FJ439578	88%	274	214/244
S7	*Atractiellales* sp. KO-2013	Basidiomycota	AB847006	100%	227	197/197
S8	*Phoma* sp. FN-40	Ascomycota	KF171356	100%	216	216/216
S9	*Monacrosporium megalosporum*	Ascomycota	AB114475	91%	270	251/276
S10	*Phoma* sp. LH128	Ascomycota	HQ832812	96%	215	208/216
S11	*Acremonium* sp. BRO-2013	Ascomycota	KF367499	92%	214	209/227
S12	*Scolecobasidium terreum* KNU9	Ascomycota	KJ921609	99%	317	293/295
