# Supplementary material for: Application of Locked Nucleic Acid (LNA) Primer and PCR Clamping by LNA Oligonucleotide to Enhance the Amplification of Internal Transcribed Spacer (ITS) Regions in Investigating the Community Structures of Plant–Associated Fungi
Source: Microbes Environ. 2016 Sep 7;31(3):339–48. doi: 10.1264/jsme2.ME16085 (PMC5017812; doi:10.1264/jsme2.ME16085)
Supplement: Supplementary file 1 [file 31_339_s1.pdf]

Supplimentary Table 1 List of LNA oligonulceotide types available for PCR clamping to inhibit the amplification of host plant DNA during PCR. The types were determined based on the sequences of agricultural plants

Plants			Accession	LNA oligonucleotide			
Family	Scientific name	Common name	number	a	b	c	other
Poaceae	***Triticum aestivum***	**Wheat**	**FJ609737**	○			
	Aegilops ventricosa	Goatgrass	ND				
	Avena sativa	Oats	ND				
	Bambusa bambos	Bamboo	GQ464805				○
	Coix lacryma-jobi	Job'S Tears	KC181920	○			
	Dactylis glomerata	Orchard grass	ND				
	Echinochloa frumentacea	Indian Barnyard Millet	KC201688	○			
	Eleusine coracana	Finger Millet	AJ272185	○			
	Festuca arundinacea	Tall fescue	ND				
	Hordeum vulgare	Barley	KJ606349	○			
	Hordeum vulgare	Barley	KJ606349	○			
	Lolium multiflorum	Italian Ryegrass	KJ598998				○
	Lolium perenne	Perennial Ryegrass	KJ598999				○
	Oryza rufipogon	Brownbeard Rice	DQ888639	○			
	Oryza sativa	Rice (Japonica)	AP009051	○			
	Oryza sativa	Rice (Indica)	FJ949064	○			
	Panicum miliaceum	Proso millet	AY129716	○			
	Phleum pratense	Timothy	ND				
	Phragmites australis	Reed	ND				
	Saccharum officinarum	Sugarcane	AB250692	○			
	Secale cereale	Rye	JF489233	○			
	Setaria italica	Foxtail Millet	KF012832.1	○			
	Sorghum bicolor	Sorghum	GQ856358				○
	Zea mays	Maize	AY097330	○			
Fabaceae	***Glycine max***	**Soybean**	**JN617195**		○		
	Arachis hypogaea	Peanut	JN617209		○		
	Astragalus sinicus	Milk vetch	ND				
	Cajanus cajan	Pigeon pea	EU288918		○		
	Lotus japonicus	Lotus	DQ311975		○		
	Medicago sativa	Alfalfa	JN617208				○
	Medicago truncatula	Barrel medic	JN617206				○
	Phaseolus coccineus	Runner bean	KF943726		○		
	Phaseolus lunatus	Lima bean	JN617200		○		
	Phaseolus vulgaris	Kidney bean	KF943720		○		
	Pisum sativum	Pea	JN617190				○
	Trifolium pratense	Red clover	JN617204				○
	Vicia faba	Broad bean	JN617193				○
	Vigna angularis	Adzuki bean	JF421525		○		
	Vigna radiata	Greem bean	KJ825885		○		
	Vigna unguiculata	Cow pea	JN617198		○		
Solanaceae	***Solanum tuberosum***	**Potato**	**LC020015**			○	
	Capsicum annuum	Redpepper	JQ885423				○
	Lycopersicum esculentum	Tomato	EU161982			○	
	Nicotiana tabacum	Tobacco	AJ012366	○			
	Solanum melongena	Eggplant	JQ638889				○
	Solanum tuberosum	Potato	LC020015			○	
Cucurbitaceae	*Citrullus lanatus*	Watermelon	JF421485	○			
	Cucumis melo	Melon	KJ467169	○			
	Cucumis sativa	Cucumber	ND				
	Cucurbita moschata	Pumpkin	ND				
	Lagenaria siceraria	Gourd	ND				
	Luffa aegyptiaca	Loofah	JN560191	○			
	Momordica charantia	Bitter Melon	KC906151	○			
Amaranthaceae	*Beta vulgaris*	Sugar beet	ND				
	Spinacia oleracea	Spinach	KF943720		○		
Brassicaceae	*Arabidopsis thaliana*	Thale cress	CP002685				○
	Brassica juncea var. *cernua*	Mustard greens	JQ085859				○
	Brassica oleracea var. botrytis	Cauliflower	ND				
	Brassica oleracea var. *capitata*	Cabbage	KF218592				○

	Brassica oleracea var. *italica*	Broccoli	ND			
	Brassica rapa var. *nippo-oleifera*	Turnip rape	GQ891874			○
	Brassica rapa var. *pekinensis*	Chinese cabbage	JN564039			○
	Brassica rapa var. *perviridis*	Komatsuna	ND			
	Raphanus sativus var. *longipinnatus*	Radish	AY746463			○
Polygonaceae	*Fagopyrum esculentum*	Buckwheat	ND			
Apiaceae	*Anethum graveolens*	Dill	AY548225	○		
	Apium graveolens var. *dulce*	Celery	AF479195	○		
	Coriandrum sativum	Coriander	KM051454	○		
	Daucus carota	Carrot	AY552527	○		
	Foeniculum vulgare	Fennel	JF421498	○		
	Petroselinum crispum	Parsley	AF479237	○		
Euphorbiaceae	*Hevea brasiliensis*	Para rubber tree	KJ665774			○
	Manihot utilissima	Cassava	GU214953			○
	Ricinus communis	Castor	KC878597			○
Araceae	*Amorphophallus konjac*	Konjac	L41383			○
	Colocasia esculenta	Eddoe	ND			
Asteraceae	*Carthamus tinctorius*	Safflower	KF886657	○		
	Cynara scolymus	Artichoke	ND			
	Helianthus annuus	Sunflower	KF767534	○		
	Lactuca sativa	Lettuce	KT074385	○		
Amaryllidaceae	*Allium cepa*	Onion	AB851493			○
	Allium chinense	Rakkyo	ND			
	Allium fistulosum	Welsh onion	JQ283850			○
	Allium sativum	Garlic	EU626375			
	Asparagus officinalis	Asparagus	ND			
Convolvulaceae	*Ipomoea batatas*	Sweet potato	JQ916066		○	
Malvaceae	*Gossypium hirsutum*	Cotton	KC404827		○	
	Hibiscus cannabinus	Kenaf	FJ527607		○	
	Hibiscus esculentus	Okra	KC404826	○		
	Theobroma cacao	Cacao	JQ228377			○
Rosaceae	*Eriobotrya japonica*	Loquat	KJ170762			○
	Fragaria x ananassa	Strawberry	X58118			○
	Malus domestica	Apple	HM235961			○
	Prunus armeniaca	Apricot	JF421472			○
	Prunus avium	cherry	HQ332167			○
	Prunus communis	Almond	KC603734			○
	Prunus mume	Japanese Plum	JF421471			○
	Prunus persica	Peach	JF421469			○
	Prunus salicina	Plum	AF179485			○
	Pyrus communis	Pear	ND			
	Pyrus pyrifolia var. *culta*	Sand pear	GU363721			○
Caricaceae	*Carica papaya*	Papaya	AF479145			○
Pedaliaceae	*Sesamum indicum*	Sesame	AF169853			○
Piperaceae	*Piper nigrum*	Pepper	KC441046			○
Cannabaceae	*Cannabis sativa*	Hemp	KC292629			○
	Humulus lupulus	Hop	AF223066			○
Zingiberaceae	*Curcuma longa*	Turmeric	KM226977			○
	Zingiber officinale	Ginger	AF205522			○
Dioscoreaceae	*Dioscorea alata*	Yam	FJ860067			○
	Dioscorea japonica	Japanese Yam	JN622212			○
	Dioscorea polystachya	Chinese Yam	FJ860110			○
	Dioscorea sansibarensis	Zanzibar Yam	DQ267929			○
Nelumbonaceae	*Nelumbo nucifera*	Lotus rhizome	FJ599761			○
Violaceae	*Vitis vinifera*	Grape	KF544886			○
Lauraceae	*Cinnamomum verum*	Cinnamon	FJ357245			○
	Persea americana	Avocado	FM957821			○
Lamiaceae	*Mentha arvensis*	Mint	KC591661		○	
Orchidaceae	*Vanilla planifolia*	Vanilla	AF391786			○
Rubiaceae	*Coffea arabica*	Coffee	EU650386		○	
Malvaceae	*Theobroma cacao*	Cacao	JQ228377			○
Arecaceae	*Cocos nucifera*	Coconut	HQ265515			○
	Elaeis guineensis	Oil palm	HQ265520			○

	Phoenix dactylifera	Date palm	ND				
Tiliaceae	*Corchorus capsularis*	Jute	FJ527599			○	
Rutaceae	*Citrus junos*	Yuzu	AB456085				○
	Citrus limon	Lemon	X05910			○	
	Citrus maxima	Pomelo	JN681156			○	
	Citrus natsudaidai	Watson pomelo	JQ990176				○
	Citrus paradisi	Grapefruit	JN681153			○	
	Citrus sinensis	Sweet orange	JN681165			○	
	Citrus unshu	Satsuma orange	JN661210			○	
Ebenaceae	*Diospyros kaki*	Persimmon	KF206042				○
Actinidiaceae	*Actinidia deliciosa*	Kiwi fruit	ND				
Lythracea	*Punica granatum*	Pomegranate	JX911356				○
Oleaceae	*Olea europaea*	Olive	JX862655				○
Juglandaceae	*Juglans mandshurica*	Walnut	HE574850				○
Fagaceae	*Castanea sativa*	Sweet Chestnut	EU016360	○			
Anacardiaceae	*Mangifera indica*	Mango	KJ833758				○
	Pistacia vera	Pistachio	KJ018019			○	
Proteaceae	*Macadamia integrifolia*	Macadamia nut	ND				
Moraceae	*Artocarpus altilis*	Breadfruit	KM234120		○		
	Artocarpus heterophyllus	Jack Fruit	JX856537		○		
	Ficus carica	Fig	EU091645		○		
	Morus bombycis	Mulberry	AY345151				
Musaceae	*Musa acuminata*	Banana	EU418631				○
Bromeliaceae	*Ananas comosus*	Pineapple	ND				
Pinaceae	*Pinus koraiensis*	Korean Pine	AY430067				○
	Pinus strobus	Eastern White Pine	AF036982				
Araliaceae	*Panax ginseng*	Korean Ginseng	KF680553	○			
Lardizabalaceae	*Akebia quinata*	Chocolate vine	JF421460				○
Cupressaceae	*Cryptomeria japonica*	Japanese cedar	U90703				○
Ephedraceae	*Equisetum arvense*	Field horsetail	ND				
Myristicaceae	*Myristica fragrans*	Nutmeg	KP406145				○
